# Precision medicine: from pharmacogenomics to pharmacoproteomics

**DOI:** 10.1186/s12014-016-9127-8

**Published:** 2016-09-26

**Authors:** Allison B. Chambliss, Daniel W. Chan

**Affiliations:** 1 Department of Pathology, Johns Hopkins University School of Medicine, Baltimore, MD 21287 USA; 2 Department of Pathology, Keck School of Medicine of USC, Los Angeles, CA 90033 USA

**Keywords:** Pharmacogenomics, Proteomics, Biomarker, Precision medicine

## Abstract

Disease progression and drug response may vary significantly from patient to patient. Fortunately, the rapid development of high-throughput ‘omics’ technologies has allowed for the identification of potential biomarkers that may aid in the understanding of the heterogeneities in disease development and treatment outcomes. However, mechanistic gaps remain when the genome or the proteome are investigated independently in response to drug treatment. In this article, we discuss the current status of pharmacogenomics in precision medicine and highlight the needs for concordant analysis at the proteome and metabolome levels via the more recently-evolved fields of pharmacoproteomics, toxicoproteomics, and pharmacometabolomics. Integrated ‘omics’ investigations will be critical in piecing together targetable mechanisms of action for both drug development and monitoring of therapy in order to fully apply precision medicine to the clinic.

## Background

Too many patients suffer from diseases with no known cure or effective treatment. Therapeutic modalities may be efficacious in some individuals or be associated with treatment failure and toxicities in others. It is well known that heterogeneities of disease presentation, genetics, and environment all contribute to the variability in drug response. With the vast improvements in technologies such as genome sequencing, big-data analysis, and electronic health records, healthcare is being revolutionized from a “one-size-fits-all” approach to a focus on the individual patient. In his 2015 State of the Union address, U.S. President Obama announced the Precision Medicine Initiative. The goal of the precision medicine approach is to integrate genetic and environmental information to have the ability to classify subpopulations of patients based on their susceptibility to particular diseases and/or their responses to particular treatments. In this manner, diagnostic testing can be used to optimize therapies for an individual. In support of this initiative, Congress recently approved the largest funding increase for biomedical research in 12 years, giving the NIH a $2 billion increase that contained $200 million for the Precision Medicine Initiative in 2016 [1]. A main mission of the initiative will be the assembly of a national research participant cohort of over 1 million Americans [2]. Data collected from the cohort will ideally allow for the identification of pharmacogenomic drug-gene relationships as well as the discovery of new biomarkers and therapeutic targets. This initiative comes at a time when technological advances have shifted biomedical studies from single genes, proteins, and metabolites to all-encompassing genomes, proteomes, and metabolomes. This review will highlight how recent progressions in both genomics and proteomics have contributed to and will continue to be simultaneously necessary for the administration of therapeutic regimens with the highest probability of success.

## Pharmacogenomics

With advances in sequencing technology and follow-ups to the Human Genome Project, such as the International HapMap Project and the 1000 Genomes Project, much of the focus for precision medicine thus far has been in the field of genomics. While a patient’s response to a drug is affected by many factors, such as drug dose, adherence and compliance to a dosing regimen, and drug–drug interactions, genetic variation in genes encoding for drug-metabolizing enzymes and transporter proteins also plays a significant role. Such genetic variation may drastically impact drug pharmacokinetics through modulation of drug absorption, distribution, metabolism, or elimination. With standard doses of implicated drugs, individuals with this type of genetic variation may experience adverse drug reactions due to drug concentrations that may either be toxic or non-efficacious. For example, single nucleotide polymorphisms (SNPs), particularly within genes encoding for the cytochrome P450 family of enzymes, have been widely implicated in aberrant drug metabolism [3].

### Pharmacogenomics approaches and applications

Genetic mutations influencing drug metabolism may be explored in a targeted single-gene manner (pharmacogenetics) or in a more global, whole-genome manner (pharmacogenomics) [4]. Single gene-drug responses, also known as candidate-gene studies, were traditionally the focus of investigations and have resulted in most of the well-established pharmacogenetic markers to date. However, improvements in broad-range sequencing technologies, including the completion of the Human Genome Project, have allowed for the increasing application of pharmacogenomic, genome-wide association studies. Such approaches allow largely unbiased investigation into genes or genetic pathways that may be involved in drug response. In vivo human experiments are ideal for these studies when they can be performed safely. Typically, patient samples are grouped by phenotype, such as patients who experienced efficacious drug effects versus no effect, or patients who experienced particular adverse reactions or toxic effects from standard doses. DNA is analyzed by broad, high-throughput sequencing approaches such as SNP microarrays, digital PCR, and next-generation sequencing. Data quality checks are critical to determine SNPs that were successfully genotyped, and appropriate allele frequency models are used to calculate accurate odds ratios and pinpoint candidate SNPs [5]. Animal studies and in vitro cell line approaches are practical alternatives for pharmacogenomic studies when drug toxicity is of concern [6, 7]. In particular, lymphoblastoid cell lines transformed with the human Epstein–Barr virus, resulting in immortalized B lymphocytes, have shown tremendous utility as a model to assess germline genetic contribution to both positive and adverse drug responses [8]. The NCI-60 cancer cell panel is also widely used to investigate the effect of somatic mutations on drug response [9].

A hallmark genome-wide drug response study in human patients was reported in 2008 by the Study of the Effectiveness of Additional Reductions in Cholesterol and Homocysteine (SEARCH) Collaborative Group, which sought to determine reasons for rare cases of statin-induced myopathy and rhabdomyolysis [10]. Assessment of over 300,000 SNP locations by bead array in patients taking equivalent doses of simvastatin revealed a strong correlation of myopathy (defined as elevated serum creatine kinase levels) with a particular SNP on the *SLCO1B1* gene, which encodes a protein involved in the hepatic uptake of various drugs. The finding led the group to further sequencing of other regions on *SLCO1B1* and resulted in the identification of several other common variants of the gene that were strongly associated with statin-induced myopathy. A recent pilot study showed that patients who received *SLCO1B1* genetically-guided statin therapy management were more likely to comply with dosing regimens and lower their LDL-cholesterol [11].

Genome-wide pharmacogenomic studies have more recently been applied in a wide variety of clinical applications, including investigations into drug concentrations and related effects of additional cholesterol- and lipid-lowering molecules [12–14], anti-depressants [15, 16], and cancer treatments [17, 18]. While most traditional cancer genomics initiatives focused on identifying the somatic mutations that drive tumor progression, it is now also recognized that germline variation among patients may significantly impact their response to cancer treatments. Initially, candidate-gene studies established associations such as *CYP2D6* polymorphisms and poor outcomes of breast cancer after tamoxifen treatment [19], or toxicity-inducing *UGT1A1* polymorphisms with irinotecan treatment of colon cancer [20]. Both of these pharmacogenetic associations have since been used to guide dosages of these drugs in a clinical setting [21, 22] and are included in expert consensus guidelines offered by the Clinical Pharmacogenetics Implementation Consortium (CPIC) [23]. Currently, there are increasing genome-wide association studies which serve to discover novel genetic biomarkers associated with variant cancer drug efficacy or toxicity in a broad, high-throughput manner. An unprecedented genome-wide study into the genetic association of variable outcomes of 5-fluorouracil and oxaliplatin (FOLFOX) treatments for colorectal cancer patients used SNP array technology to reveal seven SNPs significantly correlated with gastrointestinal, hematological, and neurological adverse drug reactions [24]. Recently, a similarly-designed study found two SNPs associated with myelosuppression in non-small cell lung cancer patients receiving platinum-based therapies [25]. Such associations would likely have never been discovered without the use of unbiased genome-wide approaches. However, functional studies are needed to further understand the pathways and mechanisms associated with these genetic aberrations in order to consider them for clinical use.

### Future directions for pharmacogenomics

Future directions for pharmacogenomics studies will likely involve incorporation of epigenetic factors such as DNA methylation and histone modification into assessment of drug response. Epigenetics may explain heterogeneities in phenotype when genotype is identical. Epigenetic modifications have largely been implicated in cancer, among other diseases, and represent an important class of drug targets [26, 27]. Indeed, DNA methylation has been shown to play a significant role in regulating the expression of members of the cytochrome P450 superfamily of enzymes, which is responsible for the metabolism of over 75 percent of commonly-prescribed pharmaceuticals [28–30]. While significant evidence exists in the literature for the epigenetic regulation of genes involved in the absorption, distribution, metabolism, and excretion (ADME) of drugs [31], the clinical relevance of this regulation remains to be seen. Combined broad-screening genomic and epigenetic studies are emerging [32] and will be critical to directly link genotypes with phenotypes. Additionally, increased accessibility to next generation sequencing will allow for the identification and analysis of novel, unique variants that may be missed by the SNP array-based methods [33]. In 2012, Price et al. described the first use of whole exome sequencing to identify novel, non-*CYP2C19* genetic variants correlated to aberrant platelet responsiveness to clopidogrel [34]. More recently, whole exome sequencing also led to genetic determinants in exceptional responders to targeted anticancer therapy of pazopanib and everolimus in advanced solid tumors [35]. Indeed, as sequencing costs continue to decrease, pharmacogenetic testing in the clinic may shift from targeted assays for specific drugs to pre-emptive, broad-scale testing models using interpretive guides from such resources as CPIC [23, 36].

## Pharmacoproteomics

While the aforementioned genomic studies have provided an abundance of advancing information and clinical utility, it is at the protein level that cellular processes are functionally regulated. Expression levels of genes and their transcripts do not necessarily correlate with corresponding protein abundance [37]. While there are an estimated 19,000 protein-coding genes in the human genome [38], it is likely that the number of proteins is near or into the millions, taking into consideration the vast opportunities for posttranslational modification that exponentially increase the diversity of the human proteome. Additionally, while DNA sequencing approaches provide static snapshots of cellular processes, the more dynamic nature of proteins makes them ideal for studying kinetic responses to drug treatments. Thus, it would be beneficial for wide-scale genomic studies to be paired with analysis of the proteome. Indeed, a major limitation of the aforementioned genome-wide associated pharmacogenomics studies is the lack of understanding of the true biological mechanisms and complete cellular pathways underlying the identified genetic associations. Precision medicine should therefore encompass both pharmacogenomics and pharmacoproteomics, a more recently-emerged field which uses proteomic technologies for drug discovery and development [39]. Notably, analysis of the translation step between genome and proteome is referred to as transcriptomics. The transcriptome may be measured by such technologies as mRNA microarrays and RNA-seq [40]. As correlation between mRNA and protein may be low, transcriptomics may also be a critical component of integrated ‘omics’ approaches [41].

The term ‘pharmacoproteomics’ was not introduced in the literature until the early 2000s [39, 42, 43], near the beginning of a rapid growth period in the general field of proteomics and its technologies. While the number of publications termed with ‘pharmacogenomics’ or ‘pharmacogenetics’ approaches well into the 10,000–20,000 range, a PubMed search in June 2016 revealed only 166 results for pharmacoproteomics, with the first having been published as a conference summary in 2002 [44]. Even today, there exists no standard definition for this branch of proteomics. This review will primarily focus on pharmacoproteomics as the use of proteomic analyses in drug discovery and development.

Although many discovered therapeutic targets enter the preclinical testing phase, the number of drugs that are eventually approved for human use is relatively miniscule, especially for oncology treatments [45]. Drug failure is often due to poor pharmacokinetic properties such as low bioavailability, poor absorption, pre-mature metabolism, or adverse side effects. Pharmacoproteomics gives us the potential to study drug mechanisms at the proteome level while at the same time investigating toxicity and resistance, or perhaps discovering new drug targets, early in the drug development process. In this manner, drugs with flawed properties can be saved from further development, while newer, better-performing drugs can be discovered and moved forward.

### Pharmacoproteomics approaches and applications

Experimental workflows in proteomics approaches to drug screening and development, like genomics approaches, may be broadly classified as targeted or global. Targeted approaches may involve affinity-based or activity-based profiling techniques, which employ chemically-engineered probes to capture proteins of interest. Detailed discussion of targeted methods can be found in several informative book chapters and review articles [46–48]. While global methods are more challenging from a bioinformatics standpoint, they are advantageous because they provide unbiased, large-scale analyses and may reveal unexpected relationships among seemingly unrelated pathways. A typical in vitro workflow involves treating cells with the drug of interest, lysing the cells, digesting proteins into peptides, and then analyzing the entire proteome by mass spectrometry techniques. Stable isotope labeling with amino acids in cell culture (SILAC) is often used for accurate quantification [49]. Protein abundance is compared across drug-treated and untreated (control) conditions in order to probe the phenotypic pathways induced by the drug. Initial studies were likely biased towards the most abundant proteins, as analytical depth suffered with earlier analytical technologies. Advances in mass spectrometry, such as sensitivity, sample preparation methods, and data analysis capabilities, now allow for the identification of over 10,000 proteins in a cell line [50, 51], though current resolutions may remain too limited to detect some low-abundance markers in blood or tissue [52]. Furthermore, protein and peptide enrichment techniques permit the assessment of post-translational modifications such as phosphorylation, as demonstrated by Klammer et al. with their identification of a protein phosphorylation signature to predict response to the antineoplastic agent dasatinib in non-small cell lung cancer cell lines [53]. Notably, protein or antibody arrays are another major technique to study proteomics that may offer increased analytical sensitivities [54]. However, because such arrays require preconceiving of the proteins to be investigated, the ‘open architecture’ of mass spectrometry may be more advantageous for global, discovery studies.

A leading study in the pharmacoproteomic field, described by Ong et al. [55], utilized quantitative proteomic analysis of SILAC-labeled cell lysates to identify specific protein interactions and targets of small molecules, including kinase inhibitors and immunophilin binders. Several investigations have since followed similar methods to identify targets of anti-cancer agents [56–58]. Results have often demonstrated that pharmaceutical compounds elicit pharmacological effects through multiple protein targets that may be unrelated by genetic sequence, highlighting the importance of the broad proteomic approach to piece together complete mechanisms [59]. Furthermore, identification of multiple protein targets may lead to novel combinatorial therapies, particularly in cancer. A recent study utilized pharmacoproteomic approaches to identify and verify combined therapy towards B cell receptor (BCR) pathways and heat shock protein 90 (Hsp90) in diffuse large B cell lymphoma [60]. Pharmacoproteomics methodologies have been applied to a variety of other disease states, such as diabetes and neurovascular disease [61]. In particular, quantitative proteomics may shed light on the interaction of small molecules with the complex blood brain barrier [62].

### Toxicoproteomics

Adverse reactions are a significant cause of drug failure in the drug development pipeline. Toxicoproteomic studies incorporate similar global protein expression technologies as described above but typically focus on either acute or chronic toxicity of the small molecule(s) in question [63]. Toxicoproteomics seeks to determine how chemical exposure modifies proteins or protein expression as a form of preclinical risk assessment. Quick and efficient identification of a molecule’s toxic effect means it may be spared from further progression down the pipeline, saving money and allowing for focus shifts to alternative molecules. Furthermore, identified protein changes may translate into new biomarkers that may be used to monitor treated patients for signatures of chemical toxicity. In terms of anticancer agents, toxicoproteomic studies aid in the detection of toxicity for ideally cancer cells only when compared to proteomic signatures of treated normal cells. A comprehensive compilation of published toxicoproteomic studies on drugs both in vitro (humans and animals) and in vivo (animals) is provided by Rabilloud and Lescuyer [64]. Analyses are sometimes targeted toward specific organ systems, often the liver or kidney [65], though it is important to consider that even the most targeted of drugs may have significant impacts system-wide. Notably, toxicoproteomic studies extend beyond the application of drug development and may be used to assess the toxic effects of other chemical agents such as environmental toxins and engineered nanomaterials.

### Pharmacometabolomics

Metabolomics involves the broad characterization of small molecule metabolites in the cell or body fluid, representing the final culmination of gene expression, protein expression, and environmental influences in order to characterize a metabolic signature of a sample or patient. In similar fashion to the aforementioned fields of study, pharmacometabolomics entails the comparison of this metabolic signature before and after drug exposure [66]. Outcomes of these studies may allow for better understanding of the mechanisms underlying heterogeneities in drug response. Approaches may be targeted toward a pathway of interest or non-targeted and may utilize a variety of technical platforms. A recent study utilized GC–MS and NMR to identify distinct sets of metabolites indicative of survival and of disease progression in serum from lung cancer patients undergoing standard chemotherapy or radiation regimens [67]. Another recent study used a targeted LC–MS/MS approach to investigate signatures of such compounds as amino acids, acylcarnitines, and lipids in serum upon neoadjuvant trastuzumab-paclitaxel treatment in HER2-positive breast cancer patients. This group found that patients with favorable response to therapy exhibited significantly higher amounts of spermidine and lower amounts of tryptophan when compared to poor responders [68]. However, larger studies are needed to clinically validate these potential biomarkers.

## Integration of pharmacogenomics and pharmacoproteomics

**Fig. 1:**
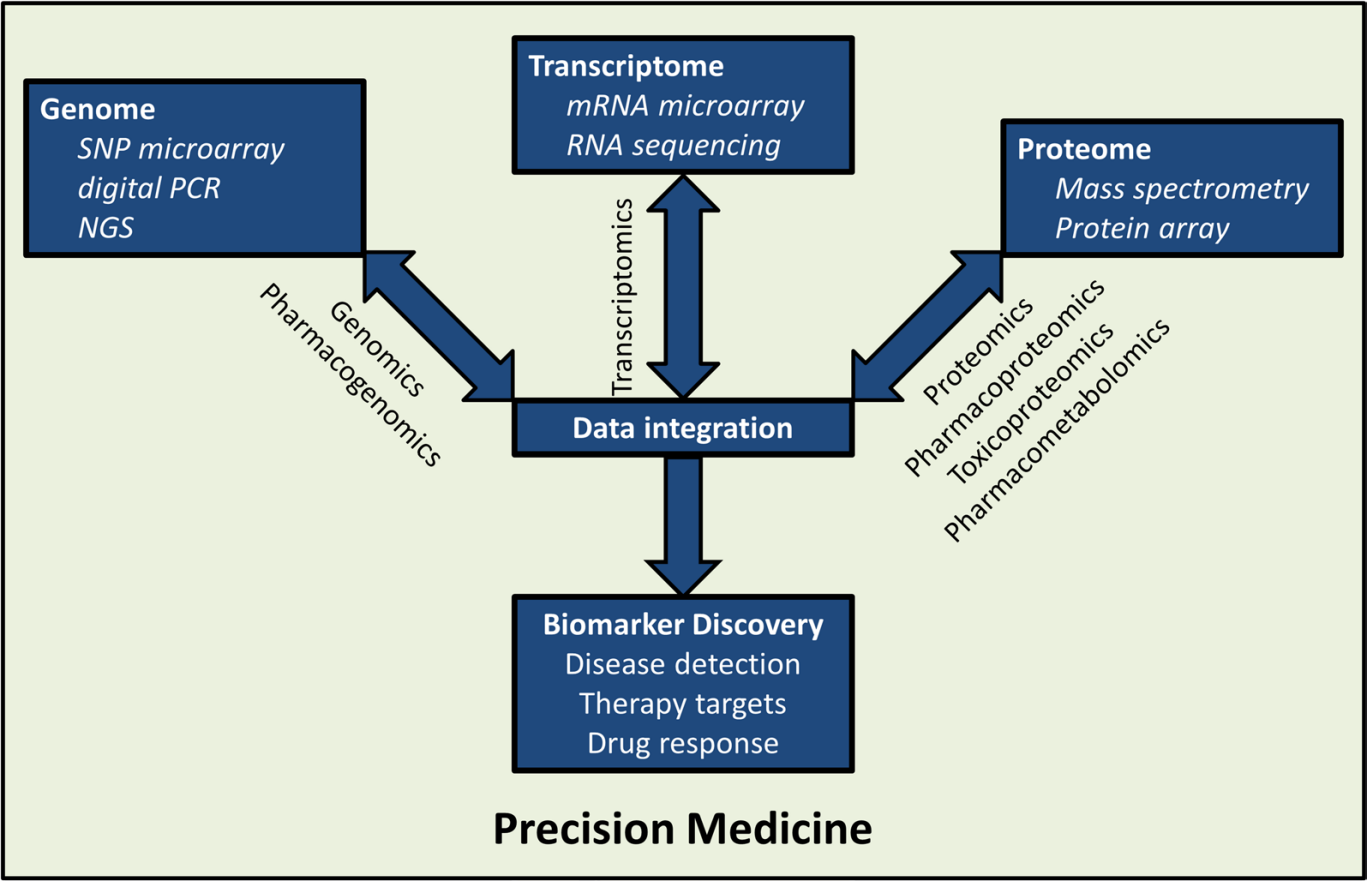
Integration of ‘omics’ technologies for precision medicine. The realization of precision medicine via the discovery and development of biomarkers for disease detection, therapy, and prediction of drug response will involve the integration of technologies which analyze control and disease-relevant samples at the genomic, transcriptomic, and proteomic levels. This schematic details some examples of such technologies. *NGS* next-generation sequencing

It is clear that research findings from the ‘omics’ fields of study, i.e. pharmacogenomics, transcriptomics, pharmacoproteomics, and associated areas of toxicoproteomics and pharmacometabolomics, should not be taken individually but instead should inform and complement one another (Fig. 1). Until recently, simultaneously-combined genomics and proteomics studies (‘proteogenomics’) had rarely been undertaken. However, advancements in systems pharmacology technologies and data management have allowed for what should be considered just the beginning of such complementing studies. One large initiative with this approach in mind is the National Cancer Institute’s Clinical Proteomic Tumor Analysis Consortium (CPTAC) [69]. The goal of the program is to identify potential cancer biomarker candidates by integrating genomic and proteomic analyses. In the “targeting genome to proteome” approach, cancer-related genome alterations first identified by genomic studies are then targeted at the protein level by proteomic measurements. In the “mapping proteome to genome” approach, broad-scale genomic and proteomic measurements are conducted simultaneously and then integrated. To date, this initiative has allowed for the unprecedented identification of protein pathways associated with genomically-annotated breast cancer samples [70] and our study on ovarian cancer samples [71]. These studies have identified novel therapeutic targets by linking genotype to phenotype, and ideally, further studies may compare the same genome and/or proteome data before and after treatment with new therapies geared toward these targets. CPTAC centers, including ours, are actively developing assays to detect and correlate candidate biomarkers. The resulting databases, as well as assay details, are posted to a free online repository in order to foster collaboration and standardization. Furthermore, in July 2016, NCI announced the launch of the Applied Proteogenomics OrganizationaL Learning and Outcomes (APOLLO) Network, a tri-agency coalition involving CPTAC, the Department of Veterans Affairs, and the Department of Defense. Through APOLLO, cancer patients will be screened for both genomic and proteomic abnormalities in order to match the patients to personalized, targeted therapies. Initially, the program will focus on a cohort of 8000 patients to investigate the genomics- and proteomics-based individualization of lung cancer treatment.

Another joint initiative stems from two additional NIH-funded multi-institution networks: the Pharmacogenomics Research Network [72] and the Pharmacometabolomics Research Network [73]. Concurrent genomic and metabolomics studies are conducted to, for instance, investigate whether genetic mutations identified as correlated with aberrant drug response are also associated with metabolites indicated for the same drug response. This approach has been coined by the networks as “pharmacometabolomics-informed-pharmacogenomics” and thus far has been applied to investigate heterogeneities in responses from pharmaceutical agents including aspirin and selective serotonin reuptake inhibitors [74]. Kaddurah-Daouk and Weinshilboum, along with the Pharmacometabolomics Research Network, provide an informative compilation of affiliated pharmacometabolomic studies in a recent review [75].

## Challenges in clinical translation

While exciting advancements are paving the way for combined ‘omics’ investigations, several barriers still remain to translate resultant biomarkers into clinical practice. Practically, challenges remain in the proteomic assessment of complex tissue and body fluids, and a majority of studies conducted to date are in vitro. Thus, identified candidates may need further verification via orthogonal, targeted approaches in more clinically-relevant matrices. Data management continues to prove challenging with the tremendous amount of data that is generated by high-throughput and broad-screening technologies. Integration of data from various experiments amongst the collaborative networks in a logistical and standardized way will be imperative in order for investigators to feed off of one another to progress discoveries.

Furthermore, once data is mined and potential biomarkers are identified, those candidate markers must be progressed forward to independent validation studies before they can be translated into the realm of clinical diagnostics. Further investigations should assess: (a) the utility of the markers in clinical outcomes studies, (b) how measurements of the markers should be interpreted and acted upon, and (c) the cost-effectiveness of implementing the monitoring of the markers. The lack of such clinical validation studies remains a significant obstacle for the implementation of pharmacogenomic and pharmacoproteomic markers alike and is a large reason that testing of many well-established pharmacogenetic polymorphisms is not widely utilized and reimbursed [36].

For similar reasons, FDA approval of assays detecting laboratory-discovered proteomic and genomic biomarkers is slow. In the case of pharmacogenomics, there is a combined desire for the FDA’s inclusion of genetic test indications on the drug label in addition to FDA approval of the corresponding genetic test itself. Progress has been made steadily, and currently more than 100 drug labels contain pharmacogenetic information specifying genetic biomarkers that may be indicated for safe use of the drug [76, 77]. As of this publication, twelve commercial nucleic acid-based assays are FDA cleared or approved in the ‘drug metabolizing enzyme’ category [78], many for *CYP2C9* for the identification of warfarin sensitivity. However, some hospital laboratories conducting pharmacogenetic testing are using laboratory-developed tests (LDTs). It remains to be seen how upcoming changes in FDA oversight of LDTs will impact pharmacogenetic testing [79, 80]. On the pharmacoproteomic side, challenges in approving proteomic biomarkers for clinical practice often include limitations in both analytical performance (i.e. precision, accuracy, sensitivity, specificity) and clinical performance, perhaps due to complexities in both sample preparation and spectra analysis methods [81, 82]. These limitations have raised issues of reproducibility with proteomics and have made harmonization of assays difficult across multiple laboratory sites. However, a recent significant multi-center study has demonstrated that standardization of both analytical and pre-analytical protocols can make possible the reproducibility of mass spectrometric measurement of proteins [83]. Further, genomic and transcriptomic technologies are currently universally considered highly reproducible [84].

## Future outlook and conclusions

Many diseases are in need of biomarker discovery of targets for treatment and monitoring and could therefore benefit tremendously from integrated ‘omics’ approaches. Cancer may be one of the most appropriate immediate focuses due to its inherent complexity and the large number of cancer genomes which have already been sequenced through collaborative efforts (i.e. the Cancer Genome Atlas [85] and the International Cancer Genome Consortium [86]). Other critical applications include the growing health problems in our nation of diabetes and metabolic syndrome. Longitudinal gathering of genomic, transcriptomic, and proteomic data among patients at-risk for these disorders may provide insight into the mechanisms behind disease progression and reveal targets for disease detection, treatment, and monitoring. Further advances in integrated data management will be critical for these studies to be successful.

As genomic and proteomic methodologies prove their analytical performance and clinical utility, become more accessible and routine, and perhaps more portable [87], one may imagine a treatment model by which such measurements may be taken at the bedside. Pharmacogenetic screening for risks of adverse drug reactions would guide drug prescription and dosing on the front end of treatment, while pharmacoproteomic measurements taken before, during, and after interventional therapy would aid in monitoring triggered phenotypic changes. Standardization of both detection methodologies and electronic healthcare databases will be critical such that patients can be followed longitudinally throughout this process.

In conclusion, personalized ‘omics’ approaches, both at the genome and proteome levels, are actively improving our understanding of disease and drug mechanisms and are allowing for the discovery, detection, and monitoring of novel biomarkers for a variety of complex diseases and their treatments. By integrating pharmacoproteomic profiles with pharmacogenomics databases, precision medicine may be eventually fulfilled via diagnosing testing to identify the right therapeutic regimen for the right patient.

## Abbreviations

SNP: single nucleotide polymorphism
SEARCH: Study of the Effectiveness of Additional Reductions in Cholesterol and Homocysteine
CPIC: Clinical Pharmacogenetics Implementation Consortium
FOLFOX: folinic acid, fluorouracil, and oxaliplatin
ADME: absorption, distribution, metabolism, and excretion
SILAC: stable isotope labeling with amino acids in cell culture
BCR: B cell receptor
Hsp90: heat shock protein 90
CPTAC: Clinical Proteomic Tumor Analysis Consortium
APOLLO: Applied Proteogenomics OrganizationaL Learning and Outcomes
